# Supplementary material for: Causal Relationship between Aging and Anorexia Nervosa: A White-Matter-Microstructure-Mediated Mendelian Randomization Analysis
Source: Biomedicines. 2024 Aug 16;12(8):1874. doi: 10.3390/biomedicines12081874 (PMC11351342; doi:10.3390/biomedicines12081874)
Supplement: Supplementary file 1 [file biomedicines-12-01874-s001.zip › Supplementary Figure/Figure S4/Singe SNP plot of MR analysis for TL to ubm-b-1678.pdf]

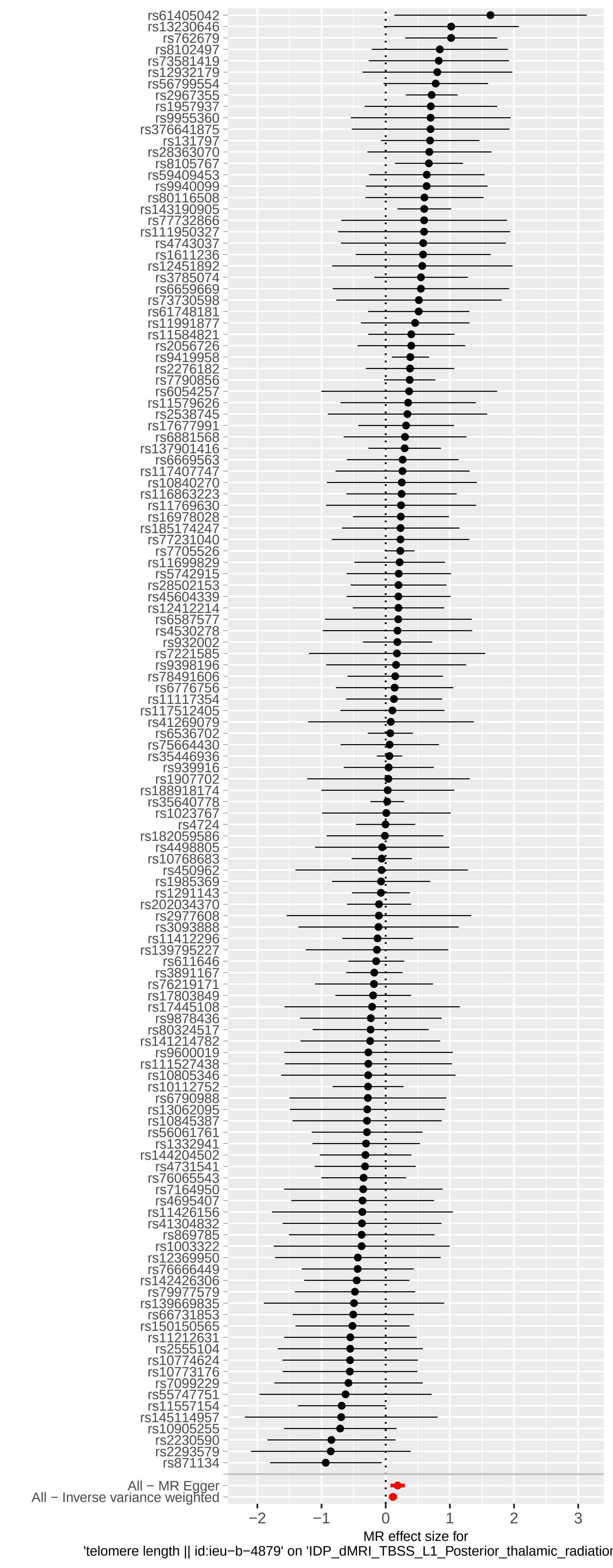
rs61405042
rs13230646
rs762679
rs8102497
rs73581419
rs12932179
rs56799554
rs2967355
rs1957937
rs9955360
rs376641875
rs131797
rs28363070
rs8105767
rs59409453
rs9940099
rs80116508
rs143190905
rs77732866
rs111950327
rs4743037
rs1611236
rs12451892
rs3785074
rs6659669
rs73730598
rs61748181
rs11991877
rs11584821
rs2056726
rs9419958
rs2276182
rs7790856
rs6054257
rs11579626
rs2538745
rs17677991
rs6881568
rs137901416
rs6669563
rs117407747
rs10840270
rs116863223
rs11769630
rs16978028
rs185174247
rs77231040
rs7705526
rs11699829
rs5742915
rs28502153
rs45604339
rs12412214
rs6587577
rs4530278
rs932002
rs7221585
rs9398196
rs78491606
rs6776756
rs11117354
rs117512405
rs41269079
rs6536702
rs75664430
rs35446936
rs939916
rs1907702
rs188918174
rs35640778
rs1023767
rs4724
rs182059586
rs4498805
rs10768683
rs450962
rs1985369
rs1291143
rs202034370
rs2977608
rs3093888
rs11412296
rs139795227
rs611646
rs3891167
rs76219171
rs17803849
rs17445108
rs9878436
rs80324517
rs141214782
rs9600019
rs111527438
rs10805346
rs10112752
rs6790988
rs13062095
rs10845387
rs56061761
rs1332941
rs144204502
rs4731541
rs76065543
rs7164950
rs4695407
rs11426156
rs41304832
rs869785
rs1003322
rs12369950
rs76666449
rs142426306
rs79977579
rs139669835
rs66731853
rs150150565
rs11212631
rs2555104
rs10774624
rs10773176
rs7099229
rs55747751
rs11557154
rs145114957
rs10905255
rs2230590
rs2293579
rs871134
All − MR Egger
All − Inverse variance weighted
−2
−1
0
1
2
3
MR effect size for
'telomere length || id:ieu−b−4879' on 'IDP_dMRI_TBSS_L1_Posterior_thalamic_radiatio
